# Prevalence of suspected COVID-19 infection in patients from ethnic minority populations: a cross-sectional study in primary care

**DOI:** 10.3399/bjgp20X712601

**Published:** 2020-09-08

**Authors:** Sally A Hull, Crystal Williams, Mark Ashworth, Chris Carvalho, Kambiz Boomla

**Affiliations:** Centre for Clinical Effectiveness and Health Data Sciences, Queen Mary University of London, London.; Centre for Clinical Effectiveness and Health Data Sciences, Queen Mary University of London, London.; Department of Primary Care and Public Health Sciences, King’s College London, London.; De Beauvoir Surgery, London.; Centre for Clinical Effectiveness and Health Data Sciences, Queen Mary University of London, London.

**Keywords:** COVID-19, ethnicity, multimorbidity, primary care

## Abstract

**Background:**

The first wave of the London COVID-19 epidemic peaked in April 2020. Attention initially focused on severe presentations, intensive care capacity, and the timely supply of equipment. While general practice has seen a rapid uptake of technology to allow for virtual consultations, little is known about the pattern of suspected COVID-19 presentations in primary care.

**Aim:**

To quantify the prevalence and time course of clinically suspected COVID-19 presenting to general practices, to report the risk of suspected COVID-19 by ethnic group, and to identify whether differences by ethnicity can be explained by clinical data in the GP record.

**Design and setting:**

Cross-sectional study using anonymised data from the primary care records of approximately 1.2 million adults registered with 157 practices in four adjacent east London clinical commissioning groups. The study population includes 55% of people from ethnic minorities and is in the top decile of social deprivation in England.

**Method:**

Suspected COVID-19 cases were identified clinically and recorded using SNOMED codes. Explanatory variables included age, sex, self-reported ethnicity, and measures of social deprivation. Clinical factors included data on 16 long-term conditions, body mass index, and smoking status.

**Results:**

GPs recorded 8985 suspected COVID-19 cases between 10 February and 30 April 2020.Univariate analysis showed a two-fold increase in the odds of suspected COVID-19 for South Asian and black adults compared with white adults. In a fully adjusted analysis that included clinical factors, South Asian patients had nearly twice the odds of suspected infection (odds ratio [OR] = 1.93, 95% confidence interval [CI] = 1.83 to 2.04). The OR for black patients was 1.47 (95% CI = 1.38 to 1.57).

**Conclusion:**

Using data from GP records, black and South Asian ethnicity remain as predictors of suspected COVID-19, with levels of risk similar to hospital admission reports. Further understanding of these differences requires social and occupational data.

## INTRODUCTION

The rapid worldwide spread of COVID-19 in early 2020, from its origin in Wuhan, China,^1^ led the World Health Organization to declare a pandemic on 11 March 2020.^2^

In the UK, early attention focused on hospital presentations and intensive care capacity, the timely supply of equipment, and, latterly, the increasing death rate in care home settings.^3^^–^^5^

Community testing, which forms part of standard public health test and quarantine policy, ceased in England on 12 March 2020,^6^ hence the extent of asymptomatic and milder symptomatic cases in community settings remains unknown. Early evidence from testing among passengers on cruise ships suggested that 18% of infected people were asymptomatic.^7^ The figures are likely to be higher in populations with a younger demographic profile.

Up to mid-April 2020, London had the highest age-standardised mortality rate for deaths in the UK reported as coronavirus, with 85.7 deaths per 100 000 population (compared with 36.6 deaths per 100 000 in England).^8^ Three of the four east London localities in this study had death rates in the top five for London boroughs (Newham = 144.3, City and Hackney = 127.4, and Tower Hamlets = 122.9 per 100 000 population).^8^ Data from the Office for National Statistics (ONS)^8^ and the OpenSAFELY^9^ study indicate that mortality rates in the most deprived areas of England were almost twice as high as those in the least deprived areas, and that males had higher death rates than females.

From an early stage in the UK epidemic, people with symptoms suggestive of COVID-19 were advised not to attend their general practice in person, and to use online or phone contact with NHS 111.^10^ Throughout general practice there was rapid uptake of technological solutions to facilitate a shift to telephone and video consultations, which enabled GPs to manage community cases, despite the national failure to share COVID-19 test results done by drive-through or home-based testing services.^11^^,^^12^ Practices worked collectively to provide separate locations for the necessary physical examinations of people with suspected COVID-19 cases and for those with other medical problems.^13^

Concern has been raised about the higher fatality rate of black, Asian, and minority ethnic (BAME) patients in intensive care units, and the disproportionate numbers of deaths of health and social care workers from these groups.^14^ Potential explanations for this greater risk include higher rates of long-term conditions such as diabetes and ischaemic heart disease among these populations, as well as housing and occupational hazards.

The population of east London includes 55% of people from minority ethnic backgrounds.^15^ Hence this geographical area is well placed to examine whether black and South Asian populations are overrepresented in the population consulting their GP practice with suspected COVID-19 symptoms, and to explore health-related causes of these differences.

**Table:** How this fits in

Patients from South Asian and black populations are at increased risk of hospital admission, intensive care admission, and death from COVID-19 infection, compared with white patients. However, little is known about the pattern of suspected COVID-19 presentations in primary care. This study found that patients of South Asian and black ethnicity are at increased risk of a clinical diagnosis of suspected COVID-19 in primary care. This risk remains even after accounting for other factors, such as multimorbidity, increasing obesity, and social deprivation, which are also strongly associated with increased risk of a suspected COVID-19 diagnosis. Primary care recording of suspected COVID-19 cases closely mirrors COVID-19 test positivity reported by the national testing scheme. Daily recording rates of suspected COVID-19 by GPs may provide an early warning system for any future upward trend in transmission rates.

The aim of this study was to identify the numbers of clinically suspected COVID-19 cases recorded by practices through the peak of the London epidemic from 10 February to 30 April 2020. It also set out to examine whether there was an excess of clinically suspected cases among the major ethnic minority groups, and how far this can be accounted for by differences in demographic status, or by differences in the burden of long-term conditions.

## METHOD

### Design and setting

This was a cross-sectional study using primary care electronic health data from 1.2 million adult patients registered at 157 general practices in the four geographically contiguous east London clinical commissioning groups (CCGs) of Newham, Tower Hamlets, City and Hackney, and Waltham Forest. In the 2011 UK census, 55% of the population in these CCGs were recorded as being of non-white ethnic origin,^15^ and the English indices of deprivation 2015 show that all four CCGs feature in the top decile of the most socially deprived boroughs in England.^16^

### Data collection

The study population included all adults (aged >18 years) registered at the 157 practices at the start of the study period, 1 January to 30 April 2020. Data were extracted on secure N3 terminals from EMIS Web, used by the majority of practices in the study area (*n* = 157/162). All data were anonymous and managed according to UK NHS information governance requirements.

Sociodemographic variables included age, sex, and self-reported ethnicity captured at the time of registration with the practice or during routine consultations. Ethnic categories were based on the 18 categories of the UK 2011 census and were combined into four groups reflecting the study population: white (British, Irish, other white), black (black African, black Caribbean, black British, other black, and mixed black), South Asian (Bangladeshi, Pakistani, Indian, Sri Lankan, British Asian, other Asian, or mixed Asian), and other (Chinese, Arab, any other ethnic group). Individuals of mixed ethnicity were grouped with their parent ethnic minority. For example, individuals who had classified themselves as mixed white and African were classed as African for the purposes of this study.^17^^,^^18^ The English indices of deprivation (IMD) 2015 score was used as a measure of social deprivation.^16^ The IMD score for each patient was mapped to the patient lower layer super output area of residence to derive internal and national quintiles for the study population.

Clinical measures included the COVID-19 SNOMED codes, which were supplied to GP computer systems from 6 February 2020.^19^ The diagnosis of suspected COVID-19 (the primary outcome measure for the study) was based on the contact history and symptoms given by patients. GPs did not have access to antigen testing during the period of the study. No results from the national testing centres were sent to GP practices.

Codes for cough, fever, upper respiratory tract infection, flu-like illness, and lower respiratory tract infection were also extracted. These may have been used for symptomatic cases before the release of the COVID-19 codes, and potentially during the course of the epidemic.

To assess the burden of long-term conditions in the study population diagnostic data were extracted on 16 conditions that form part of the UK Quality and Outcomes Framework (QOF), using the earliest recorded diagnostic code before the start of the study, based on version 44 of the QOF business rule set.^20^ The conditions included were asthma, chronic obstructive pulmonary disease, atrial fibrillation, heart failure, hypertension, coronary heart disease, peripheral arterial disease, stroke and transient ischaemic attack, chronic kidney disease, diabetes, dementia, depression, epilepsy, learning disabilities, serious mental illness, and cancer. The total count of these QOF conditions per person was used as the principal measure of multimorbidity in the adult population.^21^^,^^22^ The effect of different individual long-term conditions was explored in a sensitivity analysis.

Routine clinical data were extracted on body mass index (BMI) and smoking status as the latest recorded codes before the start of the study period. BMI values were categorised as underweight, normal, overweight, obese, and morbidly obese.

Data on daily test-confirmed COVID-19 cases done by the national testing service for England, London, and the study CCGs were obtained from the UK’s Government Digital Service website.^23^

### Statistical analysis

The primary outcome measure was prevalence of suspected COVID-19 recorded in the GP record. Statistical analysis was undertaken in Stata (version 16.1). Logistic, mixed-effect models were fitted, nesting patients within practices. Both univariate and multivariate models were fitted. The effect of ethnicity on the likelihood of suspected COVID-19 presentation was examined, adjusting for differences in demographic and clinical factors, including long-term conditions and BMI.

Sensitivity analyses were undertaken using individual comorbidities in place of counts of conditions.

## RESULTS

Primary care data from the records of 1 257 137 adult patients registered at 157 practices were available for analysis. Among this population, 8985 (0.7%) patients had a code for suspected COVID-19 in their GP record between 10 February and 30 April 2020, and 35 022 (2.8%) had a code for upper respiratory tract infection or lower respiratory tract infection between 1 January and 30 April 2020.

**Figure 1.**
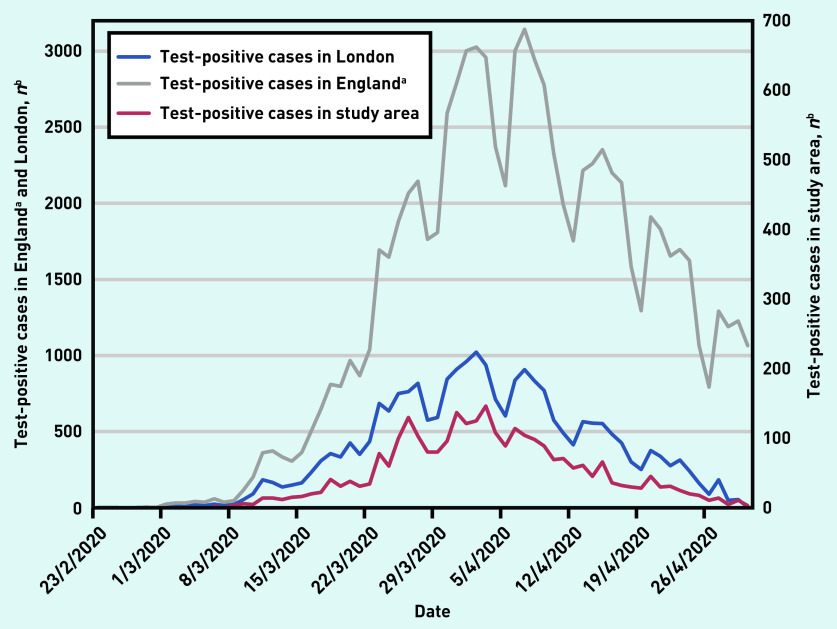
***Comparison of test-positive COVID-19 cases in the whole of England, London, and the east London study area from 23 February to 30 April 2020.*** *^a^****Testpositive cases in England are shown at 50% of total value for scaling purposes.*** *^b^****All data from the UK’s Government Digital Service website.****^23^*

Figure 1 compares the daily count of test-positive COVID-19 cases across all of England and London with those in the four study CCGs. This demonstrates that the distribution of test-positive cases in London and the study area follows a similar time course.

**Figure 2.**
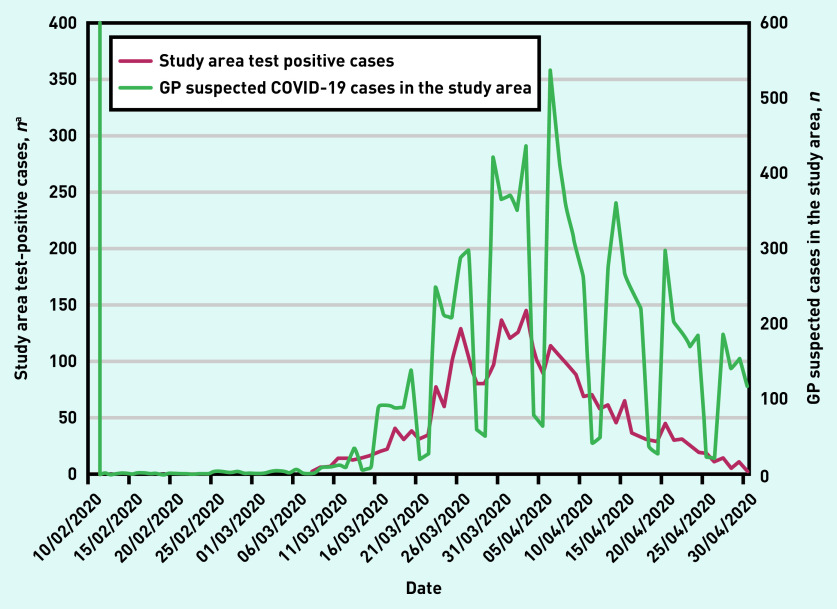
***Test-positive COVID-19 cases across the study area compared with daily counts of GP-suspected COVID-19 cases from 10 February to 30 April 2020.*** *^a^****Data on study area test-positive cases from UK’s Government Digital Service website.^23 ^Results from these positive tests were not routinely returned to general practices.***

In Figure 2, the daily count of test-positive COVID-19 cases in the study area (obtained from the UK’s Government Digital Service website^23^) is compared with suspected COVID-19 cases presenting to practices, demonstrating a similar time distribution, but three-fold greater numbers of suspected cases.

**Figure 3.**
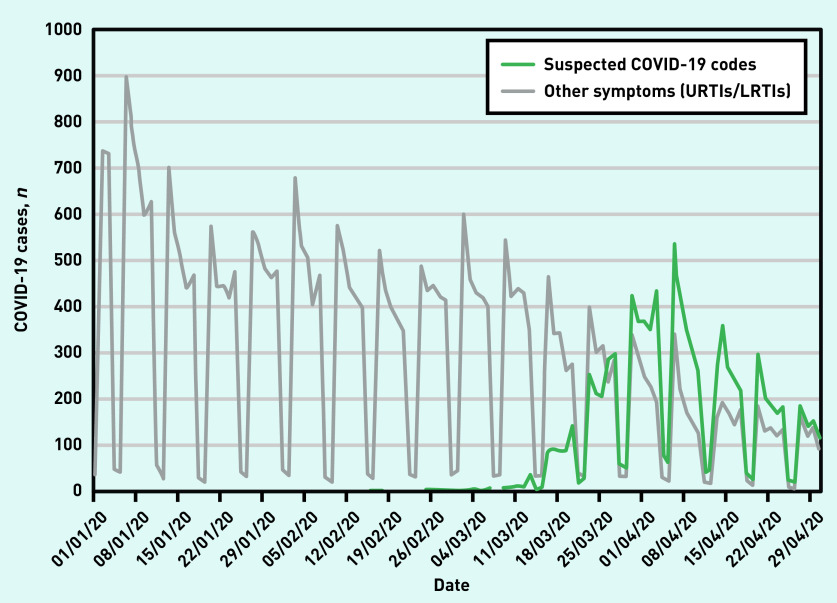
***Comparison of GP-suspected COVID-19 cases with GP-coded URTI/LRTI across the study area from 1 January to 30 April 2020.*** *^a^****Daily counts of GP-coded entries. LRTI = lower respiratory tract infection. URTI = upper respiratory tract infection.***

Figure 3 shows the daily counts of respiratory infection from 1 January to 30 April 2020 compared with the spike in suspected COVID-19 cases. This demonstrates that GPs made a clinical distinction between COVID-19 symptoms and usual upper respiratory tract infection symptoms. It also shows the seasonal decline of upper and lower respiratory tract infection cases in April, possibly magnified by social distancing.

The characteristics of patients with and without COVID-19 symptoms are shown in Table 1. Ethnicity recording was 78% complete, and 80% of cases had a valid BMI in the previous 2 years. Univariate analysis demonstrates that compared with white adults, South Asian and black adults had almost twice the odds of infection (South Asian odds ratio [OR] = 1.98, 95% confidence interval [CI] = 1.86 to 2.09; black OR = 1.88, 95% CI = 1.77 to 2.0). A total of 11.3% of adults had >1 long-term condition, and smoking prevalence was 17.4%, higher than the England average of 13.9%.^24^

**Table 1.** Characteristics of those with and without GP-suspected COVID-19 codes from 10 February to 30 April 2020 (*N* = 1 257 137 patients aged ≥18 years from 157 practices)

**Variable**	**GP-suspected COVID-19, *n* (%)**	**Without suspected COVID-19, *n* (%)**	**Univariate OR (95% CI)**
**Total**	8985	1 248 152	—

**CCG**			
Tower Hamlets	2558 (28.5)	292 653 (23.4)	—
Newham	2732 (30.4)	377 171 (30.2)	—
City & Hackney	2674 (29.8)	351 060 (28.1)	—
Waltham Forest	1021 (11.4)	227 268 (18.2)	—

**Age, years**			
18–49	5134 (57.1)	926 886 (74.3)	ref
50–69	2723 (30.3)	235 616 (18.9)	2.18 (2.08 to 2.29)
≥70	1128 (12.6)	85 650 (6.9)	2.45(2.29 to 2.62)

**Sex**			
Male	3982 (44.3)	632 082 (50.6)	ref
Female	5003 (55.7)	616 070 (49.4)	1.28 (1.22 to 1.33)

**Ethnicity**			
White	2890 (32.2)	476 302 (38.2)	ref
South Asian	2859 (31.8)	259 464 (20.8)	1.98 (1.86 to 2.09)
Black	1642 (18.3)	153 240 (12.3)	1.88 (1.77 to 2.00)
Other	594 (6.6)	78 454 (6.3)	1.24 (1.13 to 1.35)
Not stated/missing	1000 (11.1)	280 692 (22.5)	0.64 (0.60 to 0.69)

**National IMD 2015 quintiles**			
1 least deprived	30 (0.3)	8964 (0.7)	ref
2	96 (1.1)	24 029 (1.9)	1.35 (0.88 to 2.06)
3	485 (5.4)	99 395 (8.0)	1.22 (0.83 to 1.79)
4	3557 (39.6)	541 773 (43.4)	1.53 (1.05 to 2.23)
5 most deprived	4807 (53.5)	560 245 (44.9)	1.88 (1.29 to 2.74)
Missing	10 (0.1)	13 746 (1.1)	0.21 (0.10 to 0.43)

**BMI (kg/m^2^)**			
Normal weight (18.5 to <25)	2528 (28.1)	431 279 (34.6)	ref
Underweight (<18.5)	200 (2.2)	39 067 (3.1)	0.85 (0.73 to 1.00)
Overweight (25 to <30)	2770 (30.8)	299 136 (24.0)	1.60 (1.52 to 1.69)
Obese (30 to <40)	2451 (27.3)	169 982 (13.6)	2.49 (2.35 to 2.63)
Morbidly obese (≥40)	483 (5.4)	23 717 (1.9)	3.48 (3.15 to 3.84)
Out of range/Unknown	553 (6.2)	284 971 (22.8)	0.33 (0.30 to 0.36)

**QOF long-term conditions**			
0	3740 (41.6)	881 460 (70.6)	ref
1	2461 (27.4)	226 961 (18.2)	2.41 (2.29 to 2.54)
2	1350 (15.0)	81 093 (6.5)	3.75 (3.52 to 3.99)
3	690 (7.7)	33 497 (2.7)	4.60 (4.25 to 5.02)
≥4	744 (8.3)	25 141 (2.0)	6.50 (6.00 to 7.05)

**Current smoker**	1047 (11.7)	217 396 (17.4)	0.60 (0.56 to 0.63)

**Asthma**	1512 (16.8)	111 641 (8.9)	1.92 (1.81 to 2.03)

**Atrial fibrillation**	248 (2.8)	10 299 (0.8)	3.16 (2.78 to 3.59)

**Cancer**	429 (4.8)	22 989 (1.8)	2.50 (2.26 to 2.75)

**Coronary heart disease**	504 (5.6)	23 114 (1.9)	2.98 (2.72 to 3.26)

**Chronic kidney disease (stages 3–5)**	716 (8.0)	32 203 (2.6)	3.11 (2.88 to 3.37)

**COPD**	331 (3.7)	14 467 (1.2)	2.92 (2.61 to 3.26)

**Dementia**	258 (2.9)	4442 (0.4)	7.37 (6.48 to 8.39)

**Depression**	1811 (20.2)	121 290 (9.7)	2.15 (2.04 to 2.27)

**Diabetes**	1696 (18.9)	79 445 (6.4)	3.31 (3.13 to 3.49)

**Epilepsy**	157 (1.7)	10 321 (0.8)	2.00 (1.70 to 2.34)

**Heart failure**	234 (2.6)	8 039 (0.6)	3.75 (3.28 to 4.28)

**Hypertension**	2290 (25.5)	131 318 (10.5)	2.85 (2.71 to 2.99)

**Learning disability**	70 (0.8)	4660 (0.4)	1.89 (1.49 to 2.40)

**Severe mental illness**	250 (2.8)	17 322 (1.4)	1.88 (1.65 to 2.13)

**Peripheral arterial disease**	87 (1.0)	3608 (0.3)	3.00 (2.41 to 3.71)

**Stroke and TIA**	284 (3.2)	11 514 (0.9)	3.24 (2.87 to 3.65)

BMI = body mass index. CCG = clinical commissioning group. COPD = chronic obstructive pulmonary disease. QOF = Quality and Outcomes Framework. IMD = Index of Multiple Deprivation. OR = odds ratio. TIA = transient ischaemic attack.

The univariate analysis (Table 1) shows a two-fold increase in odds of suspected COVID 19 by social deprivation, with 88% of the population falling into the fourth and fifth (most deprived) national quintiles of the English IMD scores. There is a steep increase of odds associated with increasing numbers of long-term conditions and BMI categories. All long-term conditions were associated with increased odds. Although nursing and residential homes were not identified separately in this study, the sevenfold increase in risk of suspected COVID for those with dementia (OR = 7.37) may reflect the excess risk among the population of older people living in these units.

Table 2 shows the multivariate model for predictors of suspected COVID-19 for adults aged ≥18 years. This is divided into two sections, the first showing adjustment for age, sex, and social deprivation, and the second showing a fully adjusted model including the clinical predictors. For these models, internal quintiles of deprivation were used rather than national quintiles.

**Table 2.** Multivariate model for predictors of GP-suspected COVID-19 for adults aged ≥18 years (*N* = 1 257 137 patients contributing to the model)

**Variable**		**Model 1 Demographic factors**			**Model 2 Demographic and clinical factors**		
	
		**OR^a^**	**95% CI**	***P*-value**	**OR^a^**	**95% CI**	***P*-value**
**Sex**	Male	1.00	ref	ref	1.00	ref	ref
	Female	1.25	(1.20 to 1.31)	<0.001	1.17	(1.12 to 1.22)	<0.001

**Age, years**	18–49	1.00	ref	ref	1.00	ref	ref
	50–69	2.14	(2.03 to 2.24)	<0.001	1.30	(1.23 to 1.37)	<0.001
	≥69	2.57	(2.40 to 2.74)	<0.001	1.25	(1.16 to 1.35)	<0.001

**Ethnicity**	White	1.00	ref	ref	1.00	ref	ref
	South Asian	2.06	(1.94 to 2.18)	<0.001	1.93	(1.83 to 2.04)	<0.001
	Black	1.66	(1.56 to 1.77)	<0.001	1.47	(1.38 to 1.57)	<0.001
	Other	1.28	(1.17 to 1.40)	<0.001	1.41	(1.29 to 1.54)	<0.001
	Not stated/missing	0.68	(0.63 to 0.73)	<0.001	1.13	(1.05 to 1.22)	0.002

**Internal IMD 2015 quintiles^b^**	1 (least deprived)	1.00	ref	ref	1.00	ref	ref
	2	1.24	(1.14 to 1.33)	<0.001	1.18	(1.09 to 1.28)	<0.001
	3	1.23	(1.13 to 1.32)	<0.001	1.16	(1.07 to 1.25)	<0.001
	4	1.32	(1.22 to 1.43)	<0.001	1.21	(1.17 to 1.37)	<0.001
	5 (most deprived)	1.40	(1.29 to 1.51)	<0.001	1.26	(1.17 to 1.37)	<0.001

**QOF long-term conditions**	0	—	—	—	1.00	ref	ref
	1	—	—	—	1.77	(1.67 to 1.87)	<0.001
	2	—	—	—	2.28	(2.13 to 2.45)	<0.001
	3	—	—	—	2.60	(2.37 to 2.85)	<0.001
	≥4	—	—	—	3.67	(3.33 to 4.03)	<0.001

**BMI (kg/m^2^)^c^**	Normal weight (18.5 to <25)	—	—	—	1.00	ref	ref
	Underweight (<18.5)	—	—	—	0.84	(0.73 to 0.97)	0.02
	Overweight (25 to <30)	—	—	—	1.31	(1.24 to 1.38)	<0.001
	Obese (30 to <35)	—	—	—	1.73	(1.63 to 1.84)	<0.001
	Morbidly obese (>40)	—	—	—	2.23	(2.01 to 2.47)	<0.001

Intraclass correlation coefficient for practice variation is 0.16 (95% CI = 0.13 to 0.20).

a Adjusted for other variables in the table.

b Unknown IMD quintiles not shown.

c Unknown BMI not shown. BMI = body mass index. IMD = Index of Multiple Deprivation. OR = odds ratio. QOF = Quality and Outcomes Framework.

The fully adjusted model (Table 2) shows that compared with white adults, South Asian adults still had nearly twice the odds of suspected infection (OR = 1.93, 95% CI = 1.83 to 2.04), while the OR for black adults reduced to 1.47 (95% CI = 1.38 to 1.57). There is a steep gradient of odds associated with increasing numbers of long-term conditions and categories of BMI; however, these factors do not have much explanatory effect on the prevalence of suspected disease by ethnicity. The effect of social deprivation on the odds of infection was reduced in the fully adjusted model (OR = 1.26, 95% CI = 1.17 to 1.37). The fully adjusted model also shows a slight increase in risk of suspected disease for females compared with males (OR = 1.17, 95% CI = 1.12 to 1.22).

A sensitivity analysis using individual comorbidities, rather than numbers of long-term conditions, did not improve the explanatory effect of the model (see Supplementary Table S1 for details).

Consultation rates from 1 January to 31 May 2020 with GPs were examined for each ethnic group in the study. These were similar to rates during the same period in 2019, suggesting there was no surge in differential consulting related to the media-reported risks to ethnic minority populations (see Supplementary Table S2 for details).

## DISCUSSION

### Summary

Using patient-level data from the GP record, this study documents the numbers of suspected COVID-19 cases presenting to practices during the peak of the London epidemic (Figure 2). Data from these GP-suspected cases illuminate predictors of infection at an earlier stage of the disease trajectory than data from hospital or ONS case fatality reports.^8^^,^^14^

A close to two-fold increase in the odds of suspected infection for South Asian and black patients shown in the univariate analysis (Table 1) is reduced by only a small amount when adjusted by demographic and clinical factors in the multivariate analysis (Table 2). The sizeable residual risk for ethnic minority groups in the fully adjusted analysis remains unexplained.

Having a number of comorbidities, and being overweight or obese are both major independent risk factors in adult patients, but the overall effect of social deprivation was reduced in the multivariate analysis.

Figure 2 shows that GP coding for suspected COVID-19 follows the same distribution as the national data on test-positive cases, but with a three-fold greater volume, reflecting the large number of community cases. Many symptomatic individuals, following government advice, will have contacted NHS 111 rather than their GP practice. Many others with mild symptoms will have made no contact with health services, including those people who were asymptomatic. The results from viral antigen tests done either in government-run centres or in hospital settings were not routinely returned to general practice during the study period.^12^

Figure 3 shows that recorded upper and lower respiratory infection episodes fell sharply during March, during the period that saw a rise in suspected COVID-19 cases. This reflects the usual seasonal decline in viral upper respiratory tract infections, which may have been enhanced by social distancing. The national Royal College of General Practitioners (RCGP) surveillance practice data show similar trends.^25^^,^^26^ These data suggest that GPs were able to identify COVID-19 from the presenting clinical symptoms, and were able to distinguish COVID-19 symptoms from those of seasonal upper respiratory tract infections.

### Strengths and limitations

The strength of this study is the use of primary care data for the entire population registered at 157 general practices in adjacent CCGs in east London. The high level of ethnicity recording, coupled with the accurate recording of comorbidities associated with QOF, provides a unique opportunity to explore how clinical factors and demography affect the prevalence of suspected COVID-19 by ethnicity. Using UK government data on test-confirmed cases by London borough,^23^ this study confirms that GP-coded data for suspected COVID-19 follow the same time course as the London epidemic (Figures 1 and 2).

The inclusion of all episodes of upper and lower respiratory tract infections from January suggests good separation of these clinical syndromes in east London practices. Data from RCGP surveillance practices suggest that BAME populations present to GPs with upper respiratory tract infections at similar rates as the white population.^26^

Limitations common to studies using routinely collected clinical data include potential diagnostic inaccuracies, and under-recording of some conditions. GPs did not have access to COVID-19 antigen testing, hence most recorded cases reflect suspected disease. It is likely that this study underestimates the effect size, as many patients who were asymptomatic or had mild symptoms did not seek medical advice, and many patients who contacted NHS 111 (but not their practice) or went to emergency departments will fall into the population not coded for suspected COVID-19. In contrast to studies that use an extended list of comorbidities or weighted comorbidity scores,^27^ the current study used a simple count of 16 conditions in QOF, as these are well recorded across practices.^21^

It was not possible to include potentially important measures, such as household size and inter-generational composition; employment factors, including travel and activity more likely to result in exposure; or the availability of personal protective equipment. Such social and cultural factors are likely to make significant contributions to the observed differences in disease prevalence by ethnicity, but may require bespoke datasets to provide answers.

### Comparison with existing literature

The trends in risk from this study are largely consistent with the findings on ethnicity, socioeconomic status, and risk of death from COVID-19 based on hospital deaths, and with ONS reports that include deaths in hospital and community settings, adjusted by aggregate data on self-reported health and household composition (albeit these data were collected in 2011).^28^

This similarity in risk of disease for ethnic minority adults is surprising, in that this study includes milder episodes of disease, many among younger people, and mostly managed in primary care. In contrast with other studies, the current study did not find an excess of male cases, but found that females had a slight increase in risk of suspected COVID-19. This may reflect a reluctance of males to report disease at an early stage, or that sex differences only become apparent further along the disease trajectory.

The risks of disease associated with smoking have been disputed, with some studies showing lower risks of positive tests, hospital admission, or death among current smokers.^9^^,^^29^ A recent meta-analysis suggests higher risks of COVID-19 for smokers and people with COPD.^30^ The coded smoking data in the current study were limited to current smoking/non-smoking status. This may introduce bias, in that recent ex-smokers, who may stop because of respiratory symptoms or cardiovascular disease, are included among the nonsmokers. Hence smoking was not included in the multivariate analysis.

### Implications for research and practice

This study demonstrates that much of the COVID-19 epidemic is being managed in primary care, which has rapidly adjusted to requirements for consultations that are not face-to-face. Consultations in general practice may therefore be useful as an early warning system for detection and monitoring of new outbreaks of disease, which may follow the relaxation of lockdown restrictions. Practice infrastructure should be used to support testing and contact tracing. Ensuring the timely reporting of COVID-19 test results to practices, and diagnostic information from NHS 111, is a necessary part of this strategy, and will enable practices to provide continuing care to patients with more severe episodes.

Unpicking the underlying reasons for the higher risk of COVID-19 infection among those from ethnic minority populations will require studies that include data from a range of other sources, including household composition, overcrowding, and a range of factors associated with occupational exposure.
